# Comparison of long-term outcomes in simultaneous pancreas-kidney transplant versus simultaneous deceased donor pancreas and living donor kidney transplant

**DOI:** 10.1038/s41598-022-27203-w

**Published:** 2023-01-02

**Authors:** Jin-Myung Kim, Youngmin Ko, Minha Choi, Hye Eun Kwon, Jae Jun Lee, Joo Hee Jung, Hyunwook Kwon, Young Hoon Kim, Sung Shin

**Affiliations:** Division of Kidney and Pancreas Transplantation, Department of Surgery, Asan Medical Center, University of Ulsan College of Medicine, 88, Olympic-Ro 43-Gil, Songpa-Gu, Seoul, 05505 Republic of Korea

**Keywords:** Transplant immunology, Kidney diseases

## Abstract

Simultaneous deceased donor pancreas and living donor kidney transplant (SPLK) has certain advantages over conventional simultaneous pancreas-kidney transplant (SPK) and may be beneficial for overcoming the paucity of organs needed for diabetic patients requiring transplant. We compared the clinical outcomes of patients who underwent either SPK (n = 149) or SPLK (n = 46) in terms of pre- and post-transplantation variables, development of de novo DSA, occurrence of biopsy-proven acute rejection (BPAR), and graft survival rates. There were no significant differences in the baseline characteristics between the SPK and SPLK groups except for the shorter cold ischemic time of kidney grafts, shorter duration of diabetes, older age of pancreas graft-donors, and younger age of kidney graft-donors in the SPLK group. Our results showed that the death-censored pancreas graft survival rate was lower in the SPLK group. In addition, the incidence of BPAR of the pancreas graft was higher in the SPLK group. There was no significant difference in the presence of de novo DSA and the rates of kidney graft failure, kidney BPAR, and mortality. Our results show that SPLK can be considered an alternative option for SPK although higher incidences of BPAR and graft failure of pancreas after SPLK need to be overcome.

## Introduction

Diabetes mellitus (DM) is one of the main causes of various morbidities, often leading to serious complications including kidney failure. Pancreas transplantation has been utilized as a treatment modality for diabetic patients to restore the total normoglycemic state and achieve insulin independence^1^. Traditionally, pancreas transplants have been performed in various forms including simultaneous pancreas-kidney transplant (SPK), pancreas after kidney transplant (PAK), and pancreas transplant alone (PTA), which is usually reserved for those with brittle diabetes without uremia^2,3^.

SPK has been well-established as a treatment option for patients with DM and kidney failure by showing superior outcome results compared with solitary kidney transplantation in terms of kidney allograft function, patient survival, and progression of diabetic complications^4–7^. Although SPK has been the most widely performed procedure for diabetic patients with kidney failure, the waiting time for SPK is often long; according to the OPTN/SRTR 2020 annual data report, the percentage of candidates waiting for SPK continued to increase when compared with PAK or PTA^8^. To shorten the duration of dialysis, PAK may be recommended if a recipient has a living kidney donor, although this has a drawback of necessitating sequential procedures.

To overcome the paucity of organs needed for simultaneous multi-organ transplantation, simultaneous deceased donor pancreas and living donor kidney transplant (SPLK) was incorporated^9^. In this type of multi-organ transplantation, a patient receives a pancreas from a deceased donor and a kidney from a living donor. SPLK offers benefits in terms of shorter waiting time and an expanded pool of organ donors. Also, transplantation can be completed in a single operative procedure in contrast to PAK.

The purpose of this study was to compare the clinical outcomes of SPK and SPLK in diabetic patients with kidney failure in terms of patient survival, allograft survival, and biopsy-proven acute rejection of pancreas and kidney allograft.

## Results

### Baseline characteristics

The baseline characteristics of the recipients and the donors are summarized in Table 1. Duration of DM (21.1 ± 6.0 vs. 17.8 ± 7.9 years, p = 0.004) and waiting time since registration (54.9 ± 29.6 vs. 8.4 ± 12.4 months, p < 0.001) were significantly longer in the SPK recipients than in the SPLK recipients. The SPK recipients had a higher level of pre-transplant C-peptide (3.53 ± 4.72 vs. 1.98 ± 3.10 ng/mL, p = 0.012) and lower HLA mismatch (3.63 ± 1.27 vs. 4.10 ± 1.23, p = 0.027). For the SPK recipients, exocrine drainage of the pancreas allograft was performed by enteric drainage for all cases, whereas bladder drainage was performed in 78% (n = 36) of the SPLK recipients. The number of readmissions in the SPLK group and the SPK group were 6.09 ± 6.19 and 3.23 ± 3.69, respectively (p = 0.034). There were 5 patients in the SPK group and 3 patients in the SPLK group with pre-transplant DSA.

**Table 1:** Recipient and donor demographic data and baseline characteristics for SPK and SPLK groups in the study.

Variables	Overall (n = 195)	SPK (n = 149)	SPLK (n = 46)	P-value*
**Recipient characteristics**				
Age, years	41.3 ± 8.4	41.6 ± 8.5	40.2 ± 8.0	0.339
Female sex	97 (49.7)	77 (52.0)	20 (43.5)	0.311
Body mass index, kg/m^2^	21.2 ± 2.9	21.3 ± 2.7	30.9 ± 3.6	0.368
Onset of DM, years	20.9 ± 8.3	20.4 ± 8.1	22.7 ± 8.8	0.1
Duration of DM, years	20.2 ± 6.7	21.1 ± 6.0	17.8 ± 7.9	0.004
Insulin amount in use, IU/day	29.4 ± 20.9	28.2 ± 22.1	33.2 ± 16.0	0.157
HbA1c, %	7.55 ± 1.53	7.57 ± 1.59	7.49 ± 1.30	0.776
C-peptide, ng/mL	3.15 ± 4.42	3.53 ± 4.72	1.98 ± 3.10	0.012
Anti-GAD antibody, U/mL	1.86 ± 8.53	1.63 ± 7.79	2.6 ± 10.7	0.288
Bladder drainage	36 (18.4)	0 (0)	36 (78.3)	< 0.001
Presensitized patients (PRA > 20%)	52 (26.8)	44 (29.7)	8 (17.4)	0.099
HLA mismatch, n	3.56 ± 1.66	3.94 ± 1.31	3.40 ± 1.78	0.027
Kidney	–	–	2.76 ± 1.93	
Pancreas	–	–	4.07 ± 1.31	
Donor-Donor**	–	–	4.35 ± 1.32	
Number of readmissions, n	3.91 ± 4.57	3.23 ± 3.69	6.09 ± 6.19	0.034
Operating time, minutes	445.8 ± 76.2	443.2 ± 75.4	454.3 ± 78.7	0.683
Hospital days, days	31.4 ± 17.6	31.8 ± 19.1	30.0 ± 11.3	0.213
Waiting time, months	43.9 ± 33.1	54.9 ± 29.6	8.4 ± 12.4	< 0.001
**Donor characteristics**				
Age, years	30.1 ± 11.5			
Pancreas		31.7 ± 11.9	24.9 ± 8.2	< 0.001
Kidney		31.7 ± 11.9	44.0 ± 11.0	
Female sex	63 (32.5)			
Pancreas		50 (33.8)	13 (28.3)	0.119
Kidney		50 (33.8)	27 (58.7)	
Body mass index, kg/m^2^	21.8 ± 3.4			
Pancreas		22.0 ± 3.4	21.2 ± 3.4	0.722
Kidney		22.0 ± 3.4	24.4 ± 2.8	
Cold ischemic time, minutes				
Pancreas	422.3 ± 131.7	412.1 ± 132.3	455.6 ± 125.3	0.052
Kidney	277.8 ± 156.8	331.7 ± 130.0	93.7 ± 83.3	< 0.001
Cause of death, CVA	38 (19.6)	33 (22.3)	5 (10.9)	0.088
Pancreas graft weight, mg	184.6 ± 43.9	182.8 ± 44.5	190.6 ± 41.8	0.302
Kidney graft weight, mg	184.9 ± 43.2	185.8 ± 45.1	181.7 ± 36.7	0.49
**Induction regimen**				–
Antithymocyte globulin	194 (100)	148 (100)	46 (100)	–
Basiliximab	0 (0)	0 (0)	0 (0)	
**Calcineurin inhibitor**				0.428
Tacrolimus	192 (99.0)	146 (99.0)	46 (100)	
Cyclosporine	2 (1.0)	2 (1.0)	0 (0)	

*SPK* simultaneous pancreas-kidney transplant, *SPLK* simultaneous deceased donor pancreas and living donor kidney transplant, *DM* diabetes mellitus, *HLA* human leukocyte antigen, *CVA* cerebrovascular accident.

*SPK vs. SPLK/Data are mean ± standard deviation or n (%).

**Counted HLA antigens present on living kidney donor but not on the deceased pancreas donor.

In terms of donor characteristics, the age of pancreas-graft donors was older in the SPK group (31.7 ± 11.9 vs. 24.9 ± 8.2 years, p < 0.001) whereas that of kidney-graft donors was older in the SPLK group (31.7 ± 11.9 vs. 44.0 ± 11.0 years, p < 0.001). As expected, the cold ischemic time of kidney graft was longer in the SPK group than in the SPLK group (331.7 ± 130.0 vs. 93.7 ± 83.3 min, p < 0.001). The two groups did not show significant differences in other donor characteristics including the proportion of female donors, body mass index, cold ischemic time of pancreas graft, and graft weight. As an induction regimen, antithymocyte globulin was administered to all recipients. Tacrolimus was the main class of calcineurin inhibitor for both SPK and SPLK recipients. There was no significant difference between C-peptide, HbA1c and creatinine values between two studied groups in follow-up duration up to postoperative 5 years.

### Development of de novo donor-specific anti-HLA antibodies (DSA)

**Figure 1:**
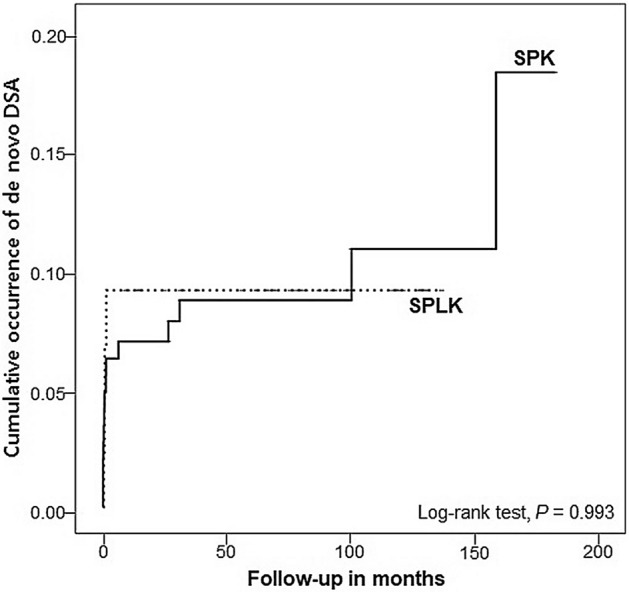
Presence of detectable de novo DSA in the study groups during follow-up. *P*-values were obtained by log-rank test statistics pairwise over strata.

Overall, anti-HLA antibodies were analyzed in all transplant patients. DSA levels were determined by the Luminex assay and quantified as mean fluorescence intensity (MFI). A total of 14 patients in the SPK group and 3 patients in the SPLK group developed de novo antibodies, although there was no significant difference in its cumulative occurrence during follow-up between the two groups (Fig. 1). Among the three patients in the SPLK group who had de novo DSA, one patient showed de novo DSA in both the kidney and pancreas grafts whereas the other two patients developed de novo DSA in only one of the grafts (one in the kidney and the other in the pancreas). The maximum MFI strength of de novo DSA in the SPK group was 15,214, and the maximum MFI strength of de novo DSA in the SPLK group with pancreas graft was 5063 and that of the SPLK group with kidney graft was 9636.

### Occurrence of BPAR and clinical rejections

**Figure 2:**
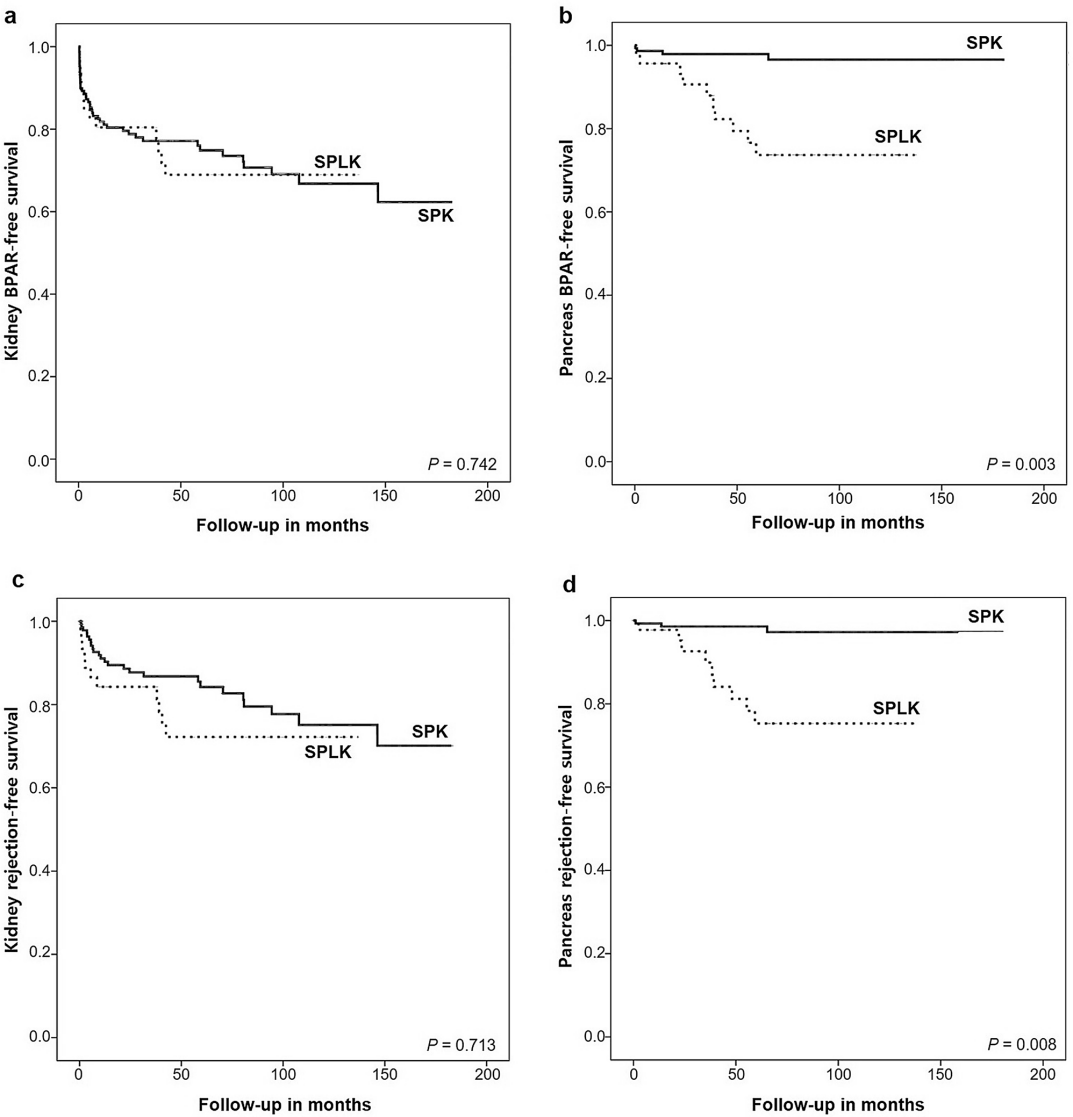
(**a**) Kaplan–Meier kidney graft BPAR free survival, (**b**) pancreas graft BPAR free survival, (**c**) kidney graft rejection-free survival, (**d**) pancreas graft rejection-free survival for simultaneous pancreas and kidney (SPK) and pancreas and living-kidney transplantation (SPLK) transplant.

The occurrence rate of BPAR of pancreas grafts was 7.7% (15/195; SPK = 5, SPLK = 10) and that of kidney grafts was 23.1% (45/195; SPK = 33, SPLK = 12). Kaplan–Meier estimated rates of kidney BPAR-free survival from the start to the end of follow-up periods were 68.9% for the SPLK group and 62.3% for the SPK group with no statistically significant difference (p = 0.742) (Fig. 2a). On the other hand, the Kaplan–Meier estimated rates of BPAR-free survival of pancreas grafts was higher in the SPK group than in the SPLK group (96.6% vs. 73.7%, p = 0.003) (Fig. 2b). Other than BPAR, we also observed a number of clinical rejections in our study. There were 16 clinical rejections of renal allograft in the SPK and 2 clinical rejections in the SPLK group. Clinical rejection of pancreas graft was seen in three patients in the SPK and one patient in the SPLK group. Rejection-free survival rates for rejection-free survival of both grafts were shown in Fig. 2c and d, showing similar results as in the BPAR-free survival rates.

### Pancreas and kidney graft survival

**Figure 3:**
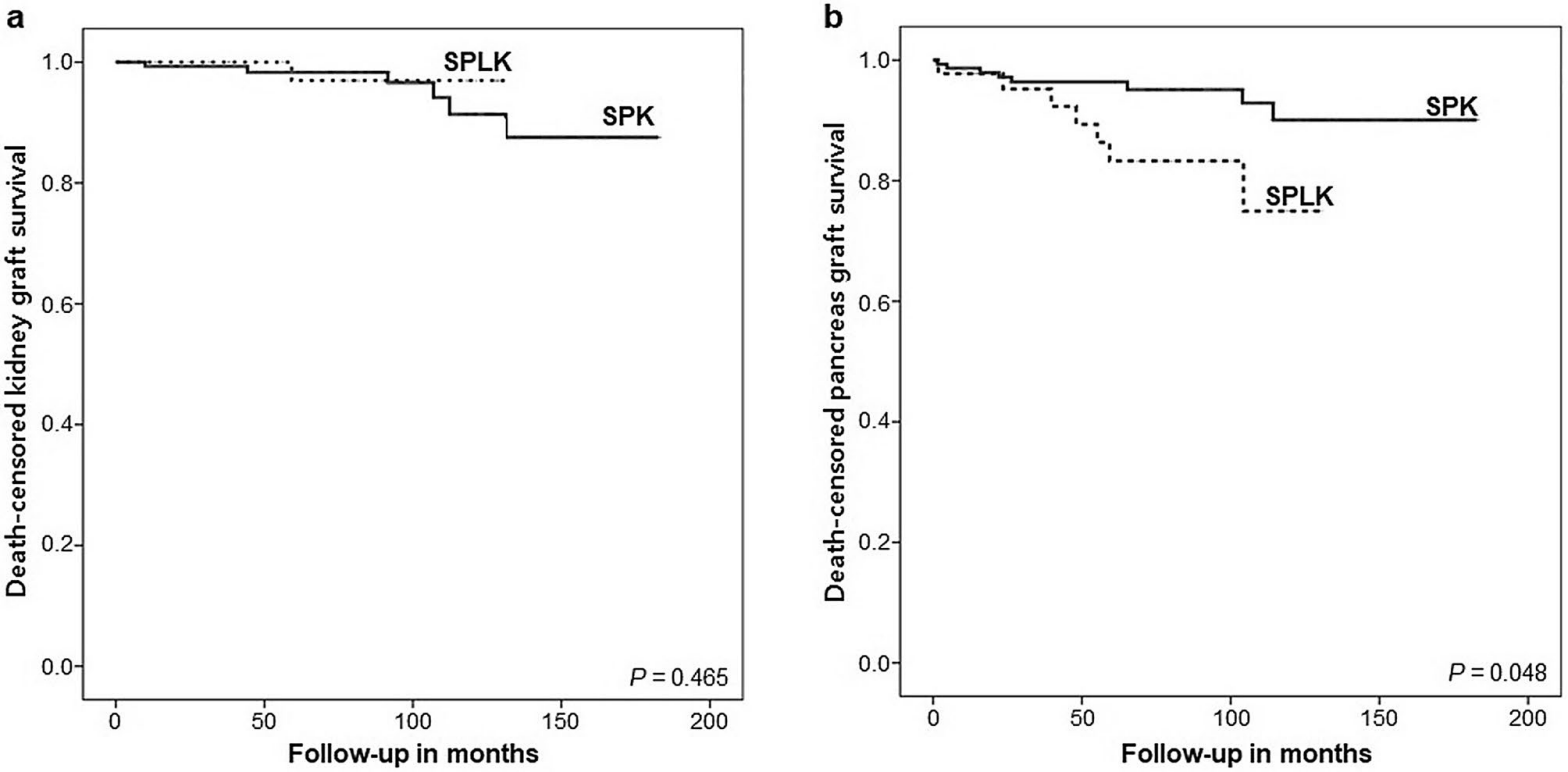
Rates of (**a**) death-censored kidney graft survival and (**b**) death-censored pancreas graft survival in the simultaneous pancreas-kidney (SPK) transplant group and the pancreas and living-kidney transplantation (SPLK) transplant group.

Kaplan–Meier survival curves showed that there was no significant difference in death-censored kidney graft survival between the two groups during follow-up; death-censored kidney graft survival rates at the end of follow-up periods were 97.0% for the SPLK group and 87.6% for the SPK group (p = 0.465) (Fig. 3a). In contrast, there was a significant difference in death-censored pancreas graft survival between the two groups; death-censored pancreas graft survival rates at the end of follow-up periods were 76.1% for the SPLK group and 90.1% for the SPK group (p = 0.048) (Fig. 3b).

## Discussion

Historically, most diabetic patients with kidney failure who desired both kidney and pancreas transplants underwent either SPK or PAK^10,11^. However, due to the lack of deceased donors and an increased number of candidates, the waiting time for SPK has become longer, thus limiting timely access to multi-organ transplantation. Moreover, PAK involves two separate operations, which can be quite a big burden for a recipient. To overcome these limitations, SPLK can be considered an alternative option in which the deceased-donor pancreas is combined with a living-donor kidney and can be performed as a single operation. The main challenge of SPLK is that it requires well-timed coordination of operations between living donor nephrectomy and deceased-donor pancreas graft recovery.

There have been only few studies that directly compared the long-term clinical outcomes between SPLK and SPK. Farney et al. reported similar one-year clinical outcomes in terms of the pancreas, kidney, and patient survival rates between SPLK and SPK recipients, while the SPLK recipients had a shorter waiting time for transplantation^12^. Our study compared death-censored graft survival as well as BPAR-free survival for both types of grafts between SPLK and SPK groups for a longer follow-up duration; although there were no statistically significant differences between the two groups in terms of death-censored survival and BPAR-free survival rates for the kidney graft, the SPK group had higher rates of BPAR-free survival and death-censored graft survival for the pancreas graft compared with the SPLK group.

In multi-organ transplantation, organs obtained from the same donor have the same immunologic background and thus the same antigenicity, which results in less production of de novo DSA compared with organs from different donors^13^. However, our results show that the number of de novo DSA was not significantly different between the two groups. Therefore, decreased pancreas graft survival rate in the SPLK group compared with the SPK group cannot be explained by the presence of newly developed DSA after transplantation. There have been several studies that analyzed the effect of de novo HLA antibodies in a simultaneous pancreas-kidney transplantation but showed conflicting results. A previous study by Malheiro et al. also showed that there was no significant association between de novo DSA and BPAR resulting in graft failure^14^, which was in agreement with our result. On the contrary, there were some previous reports that showed positive correlations between de novo* DSA* and rejections. In one paper by P.D. Uva et al.^15^ they performed protocol kidney and pancreas biopsies in de novo DSA-positive patients and found out that subclinical rejection occurred in 47% of the patients. They also asserted that detection of de novo DSA led to the decision of protocol biopsy to initiate the management in proper timing which resulted in no loss of grafts. Moreover, the presence of DSA does not always lead to an increase in graft failure; a study conducted by S. Parajuli et al.^16^ showed that the absence of rejection despite of de novo DSA presence did not result in increased risk of death-censored graft failure.

The significant difference in the rejection rate in pancreas grafts between the SPLK group and the SPK group is an interesting finding that warrants further consideration. Several reports on multi-organ transplantation including heart, lung, and kidney showed that there was a protective effect when recipients received organs from a single donor^17,18^. A recent study showed that combined heart and kidney transplantation was associated with a decrease in cardiac allograft vasculopathy by coronary plaque progression, suggesting that combined heart and kidney transplantation may have immune-modulating benefits over heart transplantation alone^19^. In addition, combined heart and lung transplantation attenuated the risk of bronchiolitis obliterans syndrome as well as cardiac allograft vasculopathy compared with single organ transplantation^20^. There was a study by O.K. Serrano et al. in literature that reported on a possible role of homologous kidney graft on pancreas allograft^21^. Similar to our results, they showed that SPLK performance in terms of the pancreas graft survival was inferior to that of SPK owing to the possible protective role of homologous kidney graft on pancreas allograft when they were from the same donors as in SPK. In addition, difficulty in the timely detection of pancreas rejection events in SPLK when compared to SPK transplant might have led to the under-treatment or delayed treatment of subclinical rejection events resulting in poorer outcomes in the SPLK group.

Our results demonstrated markedly lower overall rejection rates in terms of the pancreas graft than other previous report which reported the cumulative incidence of acute rejection as 14.7, 19.7, 26.6 and 29.1% at 1, 2, 5 and 10 years^22^. This difference in rejection rate is probably related to different postoperative management including different types of immunosuppressants during the time period covered by each study as well as practice of surveillance protocol biopsies which were not routinely performed at our center.

On the other hand, our results demonstrated higher overall kidney graft rejection rates than previously reported values of kidney graft rejections in kidney alone transplants which were less than 10%^23^. The results similar to our result was observed in another previously reported study by BN Becker et al.^24^ who assessed the impact of SPK on outcomes of type 1 diabetic patients with kidney alone transplantations (either deceased or living-related) in which SPK recipients showed significantly higher rates of renal allograft rejections compared to kidney alone transplantation patients. The reason for increase in renal allograft rejection in SPK or SPLK is not clear but we postulate that more frequent follow up for these patients caused them to perform more medical examinations compared to kidney alone transplantation contributing to higher detection of rejection events.

Our results showed that SPK recipients had less BPAR of pancreas graft than those in the SPLK group, and this might be attributed to both kidney and pancreas grafts being recovered from the same deceased donor in the SPK group. Even though the underlying mechanism is not clear, the kidney from a living donor and the pancreas from a deceased donor might have adverse immunological interactions since they have different origins. However, even though this may be the case, it is still unclear why it does not affect the kidney allograft but only the pancreas allograft. This difference should be further explored in future studies to unveil the underlying mechanism.

There is a discrepancy in reported proportion of clinical rejections in the literature. Fleiner et al. reviewed 27 publications and reported the relative proportion of clinical rejections (that is, ratio of clinical rejections over all rejection rates) with a wide range of 0–75%) in kidney transplantations^25^. The ratio of clinical rejections over all rejections (including clinical and BPAR) in our study were 28.6% (18/63) and 21% (4/19) for kidney and pancreas grafts, respectively. However, these clinical rejections did not alter the conclusive results on survival rates on both grafts as shown in Fig. 2.

Our study showed that SPLK resulted in similar rates of early graft function and survival to those of SPK. SPLK can be preferred for patients who desire to shorten their waiting time and undergo a single operation. In terms of graft functions, both SPK and SPLK have similar renal graft outcomes, which suggests that organs from different donors (e.g. pancreas from a deceased donor and kidney from a living donor) do not have an adverse effect on renal graft outcomes. However, SPLK recipients had higher incidences of BPAR and death-censored graft failure in terms of the pancreas graft when compared with SPK recipients. In conclusion, SPLK can be considered an alternative option for SPK in diabetic patients with kidney failure. We suggest that the decision between SPK and SPLK should be made carefully by weighing between shorter waiting times and poorer long-term pancreas graft outcomes.

## Methods

### Study groups, surgical procedures, and immunosuppressants

This retrospective study included 195 consecutive patients who underwent SPK (n = 149) or SPLK (n = 46) between December 2005 and June 2020 at Asan Medical Center. We excluded 9 patients who had early graft failure within 1 month postoperatively due to technical failure (n = 2; SPK = 1, SPLK = 1), massive bleeding complications (n = 6; SPK = 5, SPLK = 1), or primary non-function of the graft (n = 1, all SPK). Bleeding complications occurred immediately after the initial operations and emergency operations were performed within postoperative day 1. We further excluded 23 patients due to severe thrombosis (n = 4; SPK = 2, SPLK = 2), poor compliance (n = 1, all SPK), living-donor SPK (n = 7), severe systemic infectious disease (n = 1, all SPK) or insufficient data (n = 10; SPK = 8, SPLK = 2). Of note, the sites for thrombosis were in splenic vasculature (n = 2), in arterial graft of transplanted kidney (n = 1) and in pancreas tail artery (n = 1).

The surgical procedure for the recipient was performed as previously described^26^. To briefly describe the procedure, a midline abdominal incision was made to create a peritoneal window. Pancreas graft was situated posterior to the right colon. Vascular anastomosis for pancreas transplantation were as follows: the graft portal vein anastomosed end-to-side to the recipient's common or external iliac vein; the superior mesenteric and splenic arteries reconstructed by donor iliac arterial Y graft anastomosed to the recipient’s common iliac or external iliac artery. For enteric anastomosis, we mostly chose the distal ileum approximately 30–60 cm proximal to the ileocecal valve and used interrupted non-absorbable sutures to create a side-to-side anastomosis between the ileum and distal graft duodenum. Simultaneous kidney graft implantation was done on the contralateral side.

At our center, we implemented the method of enteric drainage for all SPK cases. One advantage of SPK is that the immunologic state of the pancreas graft could be inferred through monitoring of kidney functions since two organs were from the same donor. This allowed us to indirectly monitor the pancreas graft for possible rejections by using the kidney graft as a functional sentinel through the measurement of serum creatinine. Since grafts were from the different donors in SPLK, however, we could not implement the same strategy as in SPK. For the SPLK patients, we mainly performed bladder drainage before the year of 2018. The main advantage of bladder drainage over enteric drainage is the ability to measure urinary amylase to monitor for possible rejections as well as the avoidance of bowel-related complications such as obstruction and infection^27^. However, we had observed frequent complications associated with bladder drainage including acidosis, urinary tract infection, reflux pancreatitis, hematuria, urethritis, urethral disruption, and dysuria. Moreover, urinary amylase as a surrogate marker to detect pancreatic graft rejections was shown to be only accounting for 53% of histologically-proven rejections^28^. Therefore, we changed our center’s protocol to perform enteric drainage in all SPLK cases from 2018 onwards.

After SPK or SPLK, recipients were administered continuous intravenous heparin (400–1000 U/h). The aPTT level was measured at least every 6 h; heparin was overlapped with warfarin until the INR was reached within the therapeutic range and patients were prescribed oral warfarin for three to six months thereafter. The target levels of aPTT and PT (international normalized ratio) were 1.5 to 2 times the upper reference range.

Antithymocyte globulin was mainly administered as an induction regimen at a total dose of 4.5–5.0 mg/kg. The maintenance immunosuppressants consisted of tacrolimus, mycophenolate mofetil, and methylprednisolone for SPLK recipients whereas steroids were usually withdrawn within seven days post-transplant for SPK recipients. The target trough level of tacrolimus was 9–11 μg/L. In addition to immunosuppressants, all patients were given trimethoprim-sulfamethoxazole (80–400 mg) daily for 6 months as a prophylaxis for *Pneumocystis jirovecii* pneumonia.

The institutional review board of Asan Medical Center (Seoul, South Korea) approved this study (Approval Number: 2015-0541). The medical records were reviewed after receiving written informed consent from all studied subjects. This study was performed in accordance with the Declaration of Helsinki and all clinical activities were performed in keeping with the ethical principles outlined in the Declaration of Istanbul on Organ Trafficking and Transplant Tourism. No organs/tissues were procured from prisoners.

### Patient selection

The decision to choose between SPK and SPLK was solely dependent on the patient’s availability of a living kidney donor, reluctance to receiving multiple operations and desire to shorten their waiting time for a deceased donor regardless of their medical conditions. Therefore, there was no positive selection bias for SPLK based on geographical, clinical or immunological characteristics. When there was a living kidney donor available, the recipients were asked to choose between SPLK and PAK.

### Data collection and outcomes

Pre- and post-transplantation variables were retrospectively obtained and analyzed. Kidney graft biopsies were performed when there was an increment of more than 25% in serum creatinine values compared with the lowest creatinine value after the transplant. Pancreas biopsies were carried out when there was an evident increase in the pancreatic enzyme values in serum samples regardless of a preserved endocrine function.

### Endpoints

The primary endpoints of this study were pancreas and kidney graft survival. Pancreas graft failure was defined by a return to exogenous insulin therapy for more than 90 consecutive days. Kidney graft failure was defined as when the recipient needed to undergo persistent dialysis for 3 months or longer during the follow-up period after transplant. Secondary endpoints were biopsy-proven acute rejection (BPAR) for both pancreas and kidney grafts.

### Rejection types

In our study, if the pathologist diagnosed at least borderline rejection and we initiated antirejection treatment, the rejection was considered biopsy proven. Clinical rejection includes rejection episodes which were clinically diagnosed and medically treated while no biopsy was obtained or biopsy results did not show sufficient signs of rejection according to the Banff criteria. Detailed descriptions of each rejection including pathology results, type of rejections and treatment regimens are summarized in Supplementary Table S1 online.

### Rejection diagnosis and treatment

All biopsied tissues were stained with periodic acid-Schiff, hematoxylin and eosin, Masson trichrome, and Jones-methenamine silver. Specimens were paraffin-embedded and formalin-fixed using a commercial kit (Ventana Medical Systems, Tucson, AZ, USA). C4d immunohistochemistry (1:100, rabbit polyclonal; Cell Marque, Rocklin, CA, USA) was performed according to the manufacturer’s protocol^29^. All kidney allograft biopsy specimens were evaluated for histologic characteristics according to the Banff 2017 criteria^30^. Similar to kidney biopsies, pancreas graft biopsies were also graded for rejection based on the Banff criteria for pancreatic allograft rejection^31^. We used several parameters such as hyperglycemia, serum amylase, serum lipase, C-peptide, hemoglobin A1c to monitor the pancreas allograft function after the transplantation. Additionally, we performed additional tests such as Luminex-based single-antigen bead (SAB) immunoassay as well as imaging studies, preferably enhanced CT scan of abdomen and pelvis.

Overall, key points of our treatments for rejections for either kidney and pancreas transplants were as follows: (i) for Banff cellular rejection grade I, initial treatment was started with methylprednisolone 500 mg for 3 consecutive days and if no or subtle response was shown, anti-thymocyte globulins (ATG, 1.5 mg/kg) was added as a subsidiary treatment. (ii) for Banff cellular rejection grade II and III, steroid pulse therapy with methylprednisolone as well as T-cell depleting antibodies (either thymoglobulin 1.25 mg/kg/day or ATG 1.5 mg/kg/day) for 5 to 7 consecutive days were administered. (iii) for antibody-mediated rejection (ABMR), treatment based on intravenous immunoglobulins (IVIG) 0.5 mg/kg and plasma exchange were taken as the initial strategy. For clinically diagnosed rejections, the patients were treated with methylprednisolone 500 mg for 3 consecutive days and closely monitored for serum enzyme values. If there was no improvement, the rejections were presumed to be corticoresistant and additional treatment with T-cell-depleting antibodies as in for Banff cellular rejection grade II and II was applied. For suspicious ABMR rejections, IVIG and plasma exchange were chosen as the treatment strategy.

### HLA typing

HLA typing analysis to detect HLA-A, -B and -C exon 2,3, DRB1 exon 2 and DQB1 exon 2,3 was performed by sequence-based type using AVITA SBT Kit (Biowithus, Seoul, Korea) using a ABI PRISM 3130 Genetic Analyzer (Applied Biosystems, Foster City, CA, USA). The serum used for the HLA antibody and crossmatching (XM) test was pre-treated with dithiothreitol to inactivate the IgM antibodies. Anti-human globulin was added to enhance the amplification of the cytotoxicity reaction by complement-fixing antibodies in diluted serum and thus increasing the sensitivity of complement-dependent cytotoxicity (CDC) results. CDC XM was performed using T cells and serum derived from donors and recipients. Anti-human globulin was added to serial double dilutions of recipient sera to improve the sensitivity of CDC. CDC XM was considered positive when the observed cell death exceeded 10% above the background control value according to the American Society for Histocompatibility and Immunogenetics Laboratory Manual. Flow cytometry cross-matching (FCXM) analysis was performed using a BD FACS Canto II (BD Biosciences, San Jose, CA, USA) device. The MFI of HLA antibodies in sera was monitored using the Luminex-based SAB immunoassay. Positive T-cell FCXM and B-cell FCXM results were represented by the ratio of XM mean MFI to control MFI exceeding 2.0 and 2.5, respectively. DSA of recipient serum was measured using HLA class I and II SABs (LABScreen, One Lambda, Canoga Park, CA, USA). A Luminex system (LabScan100; One Lambda) was used to detect, acquire fluorescence data and produce data expressed as the MFI of each SAB. Patients were defined as DSA positive when MFI values  > 1000 were shown in the SAB assay.

### Statistical analysis

All data were analyzed using IBM SPSS Statistics for Windows, Version 21.0 (IBM Corp., Armonk, NY, USA). The cumulative survival outcomes of each group were examined using the Kaplan–Meier actuarial analysis and statistically compared using the log-rank test. *P* values < 0.05 were considered statistically significant. Intergroup comparisons were made in terms of the presence of de novo DSA after transplantation, biopsy-proven acute rejection of pancreas and kidney, pancreas graft failure, and kidney graft failure.

## Supplementary Information

Supplementary Table S1.

## Data Availability

The datasets used and/or analyzed in the present can be obtained on request from the corresponding author, who can be contacted at sshin@amc.seoul.kr.
