# Supplementary material for: Comparison of long-term outcomes in simultaneous pancreas-kidney transplant versus simultaneous deceased donor pancreas and living donor kidney transplant
Source: Sci Rep. 2023 Jan 2;13:49. doi: 10.1038/s41598-022-27203-w (PMC9807579; doi:10.1038/s41598-022-27203-w)
Supplement: Supplementary file 1 — Supplementary Table S1. [file 41598_2022_27203_MOESM1_ESM.docx]

**Supplementary Data for:**

**Comparison of long-term outcomes in simultaneous pancreas-kidney transplant versus simultaneous deceased donor pancreas and living donor kidney transplant**

Jin-Myung Kim^1^, Youngmin Ko^1^, Minha Choi^1^, Hye Eun Kwon^1^, Jae Jun Lee^1^, Joo Hee Jung^1^, Hyunwook Kwon^1^, Young Hoon Kim^1^, Sung Shin*

*^1^Division of Kidney and Pancreas Transplantation, Department of Surgery, Asan Medical Center, University of Ulsan College of Medicine, Seoul, Republic of Korea*

***Corresponding author:** Sung Shin

This Supplementary Data contains one supplementary table (Table S1).

Supplementary Table S1. Detailed description of all rejections observed in the study.

| **Patient#** | **Operation type** | **Rejected graft** | **Biopsy performed** | **Pathology report** | **Rejection type** | **Banff grade** | **Treatment** |
| --- | --- | --- | --- | --- | --- | --- | --- |
| 1 | SPK | Kidney | Yes | 1. Mild interstitial mononuclear cell infiltration and mild fibrosis. 2. Severe tubulitis and mild tubular atrophy. 3. Twenty eight glomeruli with mild glomerulitis, focal global sclerosis ( 1/28 ), and mild mesangial matrix increase. 4. No intimal arteritis. 5. The peritubular capillaries are focally (5%) and weakly positive on C4d immunjostaining.   : consistent with acute cellular rejection IB with mild chronic tubulointerstitial changes | aTCMR | IB | Pulse 2g |
| 2 | SPK | Kidney | Yes | 1. Mild interstitial mononuclear cell infiltration and moderate fibrosis. 2. Mild tubulitis, moderate tubular atrophy and focal cytoplasmic vacuolization. 3. Mild vascular fibrous intimal thickening, suspicious for acute cellular rejection and acute calcineurin inhibitor toxicity with moderate chronic tubulointerstitial changes.  4. The peritubular capillaries are negative on C4d immunostaining. | aTCMR | IB | Pulse 2.5g |
| 3 | SPK | Kidney | Yes | 1. Acute T-cell-mediated rejection IB. 2. Chronic active T-cell-mediated rejection. 3. Severe interstitial fibrosis and tubular atrophy. 4. Diffuse global ( 121/137 ) and segmental ( 8/137 ) glomerular sclerosis. 5. The result of BK virus immunostaining is negative. | aTCMR/caTCMR | IB | Pulse 1.5g |
| 4 | SPK | Kidney | Yes | 1. Severe interstitial mononuclear cell infiltration and moderate fibrosis 2. Severe tubulitis with and without viral cytopathic changes and moderate tubular atrophy 3. Mild vascular fibrous intimal thickening 4. A few tubular epithelial cells are positive on BK virus immunostaining.  5. The peritubular capillaries are focally ( 5% ) positive on C4d immunostaining.  : consistent with acute cellular rejection IB and polyomavirus-associated nephropathy B with moderate chronic tubulointerstitial changes | aTCMR | IB | Pulse 3g |
| 5 | SPK | Kidney | Yes | 1. Acute T-cell-mediated rejection IB. 2. C4d immunonegativity with acute and chronic microcirculation injury, suspicious for acute/active antibody-mediated rejection without evident complement deposition.  3. Severe interstitial fibrosis and tubular atrophy. 4. Diffuse global glomerular sclerosis ( 20/28 ). 5. Severe nodular arteriolar hyaline thickening, rule out chronic calcineurin inhibitor toxicity.  6. Results of immunohistochemical stainings: BK virus, (-); CD20, (+); CD56, (+) | aTCMR | IB | TPE 3 times/Pulse 2g/Rituximab 200mg |
| 6 | SPK | Kidney | Yes | 1. Acute T-cell-mediated rejection IA. 2. Chronic active T-cell-mediated rejection. 3. Mild interstitial fibrosis and tubular atrophy. 4. Focal global glomerular sclerosis ( 6/17 ). 5. Results of immunohistochemical stainings: BK virus, (-); CD20, (+); CD56, (+). | aTCMR/caTCMR | IA | Pulse 3g |
| 7 | SPK | Kidney | Yes | 1. Trivial interstitial mononuclear cell infiltration.  2. Focal tubular epithelial degeneration.  3. Four glomeruli unremarkable changes. 4. Mild vascular fibrous intimal thickening, suggestive of acute tubular injury  5. There is no evidence of acute cellular rejection and viral infection. The peritubular capillaries are focally ( 5% ) positive on C4d immunostaining. | Clinical | N/A | Pulse 1.25g |
| 8 | SPK | Kidney | Yes | 1. Trivial interstitial mononuclear cell infiltration.  2. Focal tubular epithelial degeneration.  3. Four glomeruli unremarkable changes. 4. Mild vascular fibrous intimal thickening, suggestive of acute tubular injury 5. There is no evidence of acute cellular rejection and viral  infection.  6. The peritubular capillaries are focally ( 5% ) positive  on C4d immunostaining. | Clinical | N/A | Pulse 1.5g |
| 9 | SPK | Kidney | Yes | 1. Focal global ( 13/31 ) and focal segmental ( 5/31 ) glomerular sclerosis, suggestive of focal segmental glomerulosclerosis.  2. Suspicious (Borderline) for acute T cell-mediated rejection. 3. Severe arteriolar hyalinosis, suggestive of chronic calcineurin inhibitor toxicity. 4. Moderate interstitial fibrosis and tubular atrophy. | borderline aTCMR | N/A | Thymoglobulin 5mg/kg |
| 10 | SPK | Kidney | Yes | 1. Trivial interstitial mononuclear cell infiltration. 2. Focal tubular epithelial degeneration. 3. Thirty-four glomeruli and blood vessels, unremarkable changes suggestive of acute tubular injury  4. There is no evidence of acute cellular rejection and viral infection histologically. The peritubular capillaries are negative on C4d immunostaining. | N/A | N/A | Observation |
| 11 | SPK | Kidney | Yes | 1. Acute T-cell-mediated rejection, IB. 2. Viral cytopathic changes, consistent with polyomavirus virus-associated nephropathy, I. 3. Focal tubular degeneration with isometric vacuolization, rule out acute calcineurin inhibitor toxicity. 4. Tubules are positive for BK virus and negative for CMV immunostainings, supporting the above diagnosis. The specimen includes 30 glomeruli and 3 arteries of up to intralobar size. | aTCMR | IB | Pulse 2.5g |
| 12 | SPK | Kidney | Yes | 1. C4d immunopositivity with acute and chronic microcirculation injury, suspicious for chronic active antibody-mediated rejection. 2. Suspicious (Borderline) for acute T cell-mediated rejection. 3. Severe nodular hyalinosis, suggestive of chronic calcineurin inhibitor toxicity. 4. Moderate interstitial fibrosis and tubular atrophy. 5. Diffuse global ( 9/28 ) and focal segmental ( 4/28 ) glomerular sclerosis, consistent with focal segmental glomerulosclerosis, probably secondary. | borderline caTCMR, aTCMR | N/A | TPE 4 times/Pulse 1.5g/Rituximab 100mg/ bortezomib 2 times/IVIG 400mg/kg |
| 13 | SPK | Kidney | Yes | 1. Severe tubulitis with viral cytopathic changes, consistent with polyomavirus-associated nephropathy B. 2. Focal global glomerular sclerosis ( 1/35 ) and mild chronic tubulointerstitial changes. ( See note ) | N/A | N/A | Medication stop |
| 14 | SPK | Kidney | Yes | 1. Clear cell change of tubular epithelial cells. Patchy interstitial fibrosis and tubular atrophy. 2. There is no evidence of acute rejection. Either osmotic nephrosis or acute calcineurine inhibitor toxicity should be excluded. | N/A | N/A | FK-506 dose reduction |
| 15 | SPK | Kidney | Yes | 1. Acute T-cell-mediated rejection, IA. 2. Severe interstitial fibrosis and tubular atrophy. 3. Diffuse global ( 28/32 ) and focal segmental ( 4/32 ) glomerular sclerosis. 4. One glomerulus, cellular crescent formation ( 1/32 ).  5. Results of immunohistochemical stainings: BK virus, (-); CD20, (+); CD56, (+). | aTCMR | IA | Pulse 2g |
| 16 | SPK | Kidney | Yes | 1. Mild tubular epithelial degeneration with focal isometric vacuolization, suggestive of acute calcineurin inhibitor toxicity. 2. Focal interstitial fibrosis in striped pattern, suggestive of chronic calcineurin inhibitor toxicity.  3. Focal global glomerular sclerosis ( 2/32 ) with mild interstitial fibrosis and tubular atrophy. 4. The result of BK virus immunostaining is negative. | N/A | N/A | FK-506 dose reduction |
| 17 | SPK | Kidney | No | N/A | Clinical | N/A | Pulse 2.5g |
| 18 | SPK | Kidney | Yes | 1. Chronic active T cell-mediated rejection, grade II with features of suspicious (Borderline) for acute T cell-mediated rejection. 2. Moderate interstitial fibrosis and tubular atrophy. 3. Focal global ( 17/42 ) and glomerular sclerosis ( 17/42 ). ( See note ) 4. Severe arteriosclerosis and moderate hyaline arteriosclerosis. 5. Arteriolosclerosis may present chronic calcineurin inhiloitor toxicity.  6. The result of BK virus (SV40 antigen) immunostaining is negative. | caTCMR | II | Pulse 1.5g |
| 19 | SPLK | Kidney | Yes | 1. Moderate septal lymphocytic and eosinophilic infiltration. 2. Venular endothelialitis. 3. Diffuse acinar inflammation with confluent acinar cell necrosis. 4. Markedly decreased Langerhans islets. 5. Graft fibrosis, < 30 % of biopsied area, suggestive of acute T-cell mediated rejection, grade III, with 1) recurrent autoimmune diabetes mellitus, 2) mild graft fibrosis ( chronic allograft rejection, stage 1 ).  6. Synaptophysin immunohistochemical staining confirms complete loss of Langerhans islets. 7. C4d: + in interacinar capillaries ( less than 5 % of lobular area ). Therefore, a possibility of antibody-mediated rejection is less likely. | aTCMR | III | Pulse 3g |
| 20 | SPLK | Kidney | Yes | 1. Acute T cell-mediated rejection, grade IIB.  2. Interstitial neutrophilic infiltration with neutrophilic casts and tubulitis, suggestive of acute pyelonephritis. 3. Severe interstitial fibrosis and tubular atrophy. 4. Diffuse global ( 20/33 ) glomerular sclerosis. | aTCMR | IIB | Pulse 2g |
| 21 | SPLK | Kidney | Yes | 1. Diffuse tubular degenerative changes, suggestive of acute tubular injury. 2. Focal global glomerular sclerosis ( 1/23 ). 3. Mild chronic tubulointerstitial changes.  4. The result of BK virus immunostaining is negative. | N/A | N/A | Observation |
| 22 | SPLK | Kidney | Yes | 1. Diffuse tubular degeneration, suggestive of acute tubular injury. 2. Focal global glomerular sclerosis ( 1/11 ). 3. Mild interstitial fibrosis and tubular atrophy. | N/A | N/A | Pulse 500mg |
| 23 | SPLK | Kidney | Yes | 1. Focal mild tubulitis, suspicious of acute-T-cell-mediated rejection.  2. Moderate interstitial fibrosis and tubular atrophy. 3. Moderate nodular arteriolar hyaline thickening, rule out chronic calcineurin inhibitor toxicity. 4. Focal global glomerular sclerosis ( 5/20 ). 5. The result of BK virus immunostaining is negative. | borderline aTCMR | N/A | Pulse 1.5g |
| 24 | SPLK | Pancreas | Yes | 1. Diffuse and marked septal venulitis. 2. Diffuse acinar mixed lymphoplasmocytic infiltration with occasional eosinophilic infiltration. 3. Mild intimal arteritis ( < 25 % luminal confluence ). 4. No definite C4d infiltration in interacinar capillaries. 5. Marked interstitial fibrosis ( 70 % ), consistent with acute T-cell mediate rejection, grade 3, chronic allograft rejection ( graft fibrosis ), stage 3. | aTCMR | III | Pulse 2.5g |
| 25 | SPK | Kidney | Yes | 1. Suspicious (Borderline) for acute T cell-mediated rejection. 2. Severe interstitial fibrosis and tubular atrophy. 3. Focal global ( 7/18 ) and focal segmental ( 5/18 ) glomerular sclerosis, consistent with focal segmental glomerulosclerosis, probably secondary.  4. The result of BK virus (SV40 antigen) immunostaining is negative. | borderline aTCMR | N/A | Thymoglobulin 3.1mg/kg |
| 26 | SPLK | Pancreas | Yes | 1. Septal and diffuse interstitial fibrosis ( up to 50 % ). 2. Moderate lymphocytic and eosinophilic lobular inflammation. 3. A few arteritis. 4. Equivocal C4d positive interacinar capillaries, consistent with acute T-cell mediated rejection, grade II/ moderate, chronic allograft rejection, stage II. 5. No definite antibody-mediated rejection. | aTCMR | II/II | Pulse 2g/thymoglobulin 6mg/kg |
| 27 | SPK | Kidney | Yes | 1. Mild tubulitis, suspicious of acute T-cell-mediated rejection. 2. Mild interstitial fibrosis and tubular atrophy. 3. Focal isometric vacuolization of tubular epithelial cells, rule out acute calcineurin inhibitor toxicity. 4. No glomerular sclerosis.  5. The result of BK virus immunostaining is negative. | borderline aTCMR | N/A | Pulse 1g |
| 28 | SPK | Kidney | Yes | 1. Suspicious (Borderline) for acute T cell-mediated rejection. 2. Severe interstitial fibrosis and tubular atrophy. 3. Diffuse global and glomerular sclerosis ( 32/35 ). 4. The result of BK virus (SV40 antigen) immunostaining is negative, supporting the diagnosis. | N/A | N/A | Pulse 1.5g |
| 29 | SPLK | Kidney | Yes | 1. Mild tubulitis, suspicious of acute T-cell-mediated rejection. 2. C4d immunonegativity with acute microcirculation injury, suspicious for acute/active antibody-mediated rejection without evident complement deposition.  3. Chronic active T-cell-mediated rejection. 4. Moderate interstitial fibrosis and tubular atrophy. ( See note ) 5. Focal global glomerular sclerosis ( 2/18 ).  6. The result of BK virus immunostaining is negative. | aTCMR/caTCMR/aABMR | N/A | TPE 3 times/Pulse 2g/Rituximab 200mg |
| 30 | SPK | Kidney | Yes | 1. Severe tubulitis with viral cytopathic changes, consistent with polyoma virus-associated nephropathy B. 2. Focal global glomerular sclerosis ( 2/19 ). 3. No interstitial fibrosis and tubular atrophy. ( See note ) 4. The result of BK virus immunostaining is positive. | N/A | N/A | Immunosuppressant dose reduction |
| 31 | SPK | Kidney/Pancreas | Yes | 1. Septal mononuclear cell infiltration, moderate with ductitis and venular endothelialitis. 2. Frequent spotty acinar cell injury and drop out, suggestive of acute T-cell-mediated rejection, moderate ( grade II ) 3. Multifocal C4d positivity in pancreatic lobules. 4. Circulating donor-specific antibody, consistent with acute/active antibody-medicated rejection, grade II. 5. Expansion of fibrous septa ( < 30 % ) of core surface, consistent with chronic allograft rejection, stage I ( mild graft fibrosis ). | aTCMR/aABMR | II/II | TPE 4 times/Pulse 3g/Rituximab 100mg |
| 32 | SPLK | Kidney | Yes | 1. Mild septal inflammation. 2. Focal septal venulitis and ductulitis. 3. Mild acinar inflammation. 4. No C4d staining in interacinar capillaries. 5. Minimal fibrosis, consistent with mild acute T-cell mediated rejection ( grade I ) | aTCMR | I | Thymoglobulin 4.5mg/kg |
| 33 | SPLK | Kidney | Yes | 1. Significantly decreased Langerhans islets. ( See note #1 ) 2. No significant septal inflammation. ( See note #2 ) 3. No significant acinar inflammation or damage. 4. No definite ranular endothelialitis or ductitis. 5. Mild septal fibrosis ( < 10 % ). 6. Synaptophysin, chromogranin, and insulin stainings reveal markedly shrunken Langerhans islets, suggesting selective islet cell injury. 7. There is no evidence of significant acute T-cell mediated rejection. | N/A | N/A | Observation |
| 34 | SPLK | Kidney | No | N/A | Clinical | N/A | Pulse 2g |
| 35 | SPK | Kidney | Yes | 1. Severe tubulitis with viral cytopathic changes, consistent with polyoma virus-associated nephropathy B. 2. No global glomerular sclerosis. 3. No interstitial fibrosis and tubular atrophy. ( See note ) 4. The result of BK virus immunostaining is positive. | N/A | N/A | Leflunomide |
| 36 | SPLK | Pancreas | Yes | 1. Mild acinar inflammation with acinar edema ( 2 foci/10 bule ). 2. Mild septal inflammation. 3. No venular endotheliitis or ductitis. 4. No significant fibrosis, consistent with acute T-cell mediated rejection, grade I. 5. C4d staining was noted in a few arterioles. 6. No C4d staining was observed in interacinar capillaries. | aTCMR | I | Observation |
| 37 | SPK | Kidney | Yes | 1. C4d staining without evidence of rejection. 2. Mild interstitial fibrosis and tubular atrophy. 3. Focal global ( 4/11 ) and focal segmental ( 5/11 ) glomerular sclerosis ( 9/11 ). ( See note ) 4. Diffuse mesangial proliferative glomerulonephritis. | N/A | N/A | Observation |
| 38 | SPK | Kidney | Yes | 1. Suspicious (Borderline) for acute T cell-mediated rejection.  2. Severe interstitial fibrosis and tubular atrophy. 3. Focal global ( 8/24 ) and focal segmental ( 2/24 ) glomerular sclerosis, consistent with focal segmental glomerular sclerosis, probably secondary.  4. The result of BK virus (SV40 antigen) immunostaining is negative. | borderline aTCMR | N/A | Pulse 1.5g |
| 39 | SPK | Kidney | Yes | 1. Acute T-cell-mediated rejection, IB. 2. C4d immunonegativity with severe acute microcirculation injury, suspicious for acute/active antibody-mediated rejection without evident complement deposition. 3. No glomerular sclerosis. 4. No interstitial fibrosis and tubular atrophy. | aTCMR/aABMR | IB | Pulse 2.5g |
| 40 | SPK | Kidney | Yes | 1. Suspicious (Borderline) for acute T cell-mediated rejection. 2. Mild interstitial fibrosis and tubular atrophy. 3. Focal global glomerular sclerosis ( 2/27 ) and no crescent formation. ( See note ) 4. There are diffuse thickening of glomerular basement membrane based on EM study. It could be diabetic nephropathy, class I.  5. The result of BK virus (SV40 antigen) immunostaining is negative, supporting the diagnosis. | borderline aTCMR | N/A | Pulse 1.5g |
| 41 | SPLK | Pancreas | Yes | 1. Moderate mixed septal inflammation. 2. Moderate ductitis. 3. Moderate venular endothelialitis. 4. Occasional spotty acinar injury ( up to 2 foci/ lobule ), consistent with acute cell-mediated rejection, mild (grade I). 5. Mild mixed monocytic and neutrophilic infiltration. 6. Focal C4d labeling in intraacinar capillaries. 7. Donor specific antibody: positivity, consistent with acute antibody-mediated rejection, mild ( grade I). | aTCMR/aABMR | I | TPE 3 tiems/Pulse 2.5g/Rituximab 200mg/Thymoglobulin 13.3mg/kg |
| 42 | SPLK | Kidney | Yes | 1. Mild tubulitis, suspicious of acute T-cell-mediated rejection. 2. Severe interstitial fibrosis and tubular atrophy. 3. Focal global glomerular sclerosis ( 3/28 ). 4. Results of immunohistochemical stainings: BK virus, (-); CD20, (-); CD56, (-). | borderline aTCMR | N/A | Pulse 1g |
| 43 | SPLK | Kidney | Yes | 1. Proteinous tubular casts and mild tubulitis, suggestive of obstruction. 2. Mild interstitial fibrosis and tubular atrophy. 3. Minimal glomerular lesion with weak mesangial IgA deposition suggestive of early IgA nephropathy (Haas classification, subclass I; Oxford classification, M0 E0 S0 T0 ). 4. No glomerular sclerosis ( 0/16 ). ( See note ) 5. The result of BK virus immunostaining is negative. | N/A | N/A | Observation |
| 44 | SPLK | Kidney | Yes | 1. Acute T-cell-mediated rejection IA. 2. Focal isometric vacuolization of tubules, rule out acute calcineurin inhibitor toxicity. 3. Trivial interstitial fibrosis and tubular atrophy. 4. No glomerular sclerosis. ( See note ) 5. The result of BK virus immunostaining is negative. CD56, (-); CD20, (-).negative. CD56, (-); CD20, (-). | aTCMR | IA | Pulse 2.5g |
| 45 | SPK | Kidney | Yes | 1. Chronic active T cell-mediated rejection, grade II with features of suspicious (Borderline) for acute T cell-mediated rejection. 2. C4d immunonegativity with acute microcirculation injury, rule out active antibody-mediated rejection.  3. Mild interstitial fibrosis and tubular atrophy. 4. Focal global glomerular sclerosis ( 3/19 ). ( See note ) 5. The result of BK virus (SV40 antigen) immunostaining is negative. | caTCMR/aABMR | II | TPE 3 times/Thymoglobulin 1.8mg/kg |
| 46 | SPLK | Kidney | Yes | 1. Chronic active T cell-mediated rejection, grade IA. 2. Arteriolar nodular hyalinosis and chronic tubulointerstitial changes in stripped pattern, suggestive of chronic calcineurin inhibitor toxicity. 3. A few polymorphonuclear leukocyte casts, rule out acute bacterial infection. 4. Moderate interstitial fibrosis and tubular atrophy. 5. Focal global ( 10/24 ) and focal segmental ( 8/24 ) glomerular sclerosis, consistent with focal segmental glomerulosclerosis, probably secondary. 6. The result of BK virus (SV40 antigen) immunostaining is negative. 7. The chronic active T cell-mediated rejection, grade IA includes features of acute T cell-mediated rejection, grade IA. | caTCMR | IA | Pulse 1.5g |
| 47 | SPK | Kidney | No | N/A | Clinical | N/A | TPE 4 times/Pulse 3g/Rituximab 200mg |
| 48 | SPK | Kidney | No | N/A | Clinical | N/A | Pulse 3g |
| 49 | SPLK | Pancreas | Yes | 1. Moderate septal inflammation with venular endothelialitis. 2. Occasional spotty acinar inflammation ( 1 or 2 foci / lobule ). 3. Focal ductitis, consistent with acute T-cell mediated rejection, grade I. 4. No evidence of acute antibody-mediated rejection. ( See note ) 5. No significant fibrosis. | aTCMR | I | Pulse 2g |
| 50 | SPLK | Kidney | Yes | 1. Mild tubulitis, suspicious of acute T-cell-mediated rejection. 2. Severe interstitial fibrosis and tubular atrophy, suggestive of polyomavirus nephropathy, stage C. 3. Diffuse global glomerular sclerosis ( 13/13 ). 4. Results of immunohistochemical stainings: BK virus, (-); CD20, (+); CD56, (+). | borderline aTCMR | N/A | Pulse 3g |
| 51 | SPK | Kidney | No | N/A | Clinical | N/A | Pulse 2.5g |
| 52 | SPLK | Kidney | No | N/A | Clinical | N/A | Pulse 2.5g, PP |
| 53 | SPK | Kidney | Yes | 1. Isolated V-lesion. (The isolated V-lesion may represent T-cell-mediated rejection IIA or less likely C4d-negative antibody-mediated rejection) 2. Mild tubulitis, suspicious of acute T-cell-mediated rejection. 3. Chronic active T-cell-mediated rejection. 4. No glomerular sclerosis ( 0/56 ). 5. Trivial interstitial fibrosis and tubular atrophy. ( See note ) 6. The result of BK virus immunostaining is negative. | aTCMR/caTCMR | IIA | Observation |
| 54 | SPK | Kidney | Yes | 1. Suspicious (Borderline) for acute T cell-mediated rejection. 2. Neutrophilic cast, suggestive of acute pyelonephritis. 3. Moderate interstitial fibrosis and tubular atrophy. 4. Focal global glomerular sclerosis ( 5/62 ).  5. The result of BK virus (SV40 antigen) immunostaining is negative. | borderline aTCMR | N/A | Antibiotics |
| 55 | SPK | Kidney | No | N/A | Clinical | N/A | Pulse 2g |
| 56 | SPLK | Pancreas | Yes | 1. Minimal acinar inflammation. 2. Mild septal venulitis. 3. No intimal arteritis. 4. C4d-positivity in interacinar capillaries ( 10 % ) consistent with acute antibody-mediated rejection, grade I. 5. No fibrosis. | aABMR | I | TPE 4 times/Pulse 750mg/Rituximab 100mg/IVIG 400mg/kg, |
| 57 | SPK | Kidney | Yes | 1. Acute T-cell-mediated rejection, IB. 2. No glomerular sclerosis ( 0/40 ). 3. No interstitial fibrosis and tubular atrophy. ( See note ) 4. The result of BK virus immunostaining is negative.  5. Results of immunohistochemical stainings: CD20, (-); CD56, (+). | aTCMR | IB | Pulse 2.5g |
| 58 | SPLK | Kidney | Yes | 1. Focal intimal arteritis, consistent with acute T-cell-mediated rejection IIA. 2. Focal viral cytopathic changes with BK virus immunopositivity, consistent with polyoma virus-associated nephropathy. 3. Mild interstitial fibrosis and tubular atrophy. 4. Mesangial IgA immunopositivity, suggestive of IgA nephropathy. 5. No global or segmental glomerular sclerosis. 6. Results of immunohistochemical stainings: BK virus, (+); CD20, (+); CD56, (+). | aTCMR | IIA | Pulse 2.5g |
| 59 | SPK | Kidney | Yes | 1. Acute tubular injury. 2. No evidence of active rejection. 3. Mild interstitial fibrosis and tubular atrophy. 4. Focal global glomerular sclerosis ( 7/17 ). ( See note ) 5. The result of BK virus (SV40 antigen) immunostaining is negative. | N/A | N/A | Antibiotics |
| 60 | SPLK | Kidney/Pancreas | Yes | 1. Positive donor specific antibody. 2. Septal venulitis. 3. No definite acinar cell inflammation. 4. Patchy C4d positive-intra-acinar capillaritis ( ≥ 5 % of acinar lobular surface ), consistent with grade I/mild acute antibody mediated rejection. | aABMR | I | TPE 3 times/Pulse 1.5g/Rituximab 100mg |
| 61 | SPK | Kidney | Yes | 1. Acute pyelonephritis. 2. Mild tubulitis, suspicious of acute T-cell-mediated rejection. 3. Mild interstitial fibrosis and tubular atrophy. 4. Results of immunohistochemical stainings: BK virus, (-); CD20, (+); CD56, (+). | borderline aTCMR | N/A | Pulse 750mg |
| 62 | SPK | Kidney | Yes | 1. No evidence of acute rejection. 2. Severe nodular arteriolar hyalinosis, suggestive of chronic calcineurin inhibitor toxicity. 3. Mild interstitial fibrosis and tubular atrophy. 4. Focal global glomerular sclerosis ( 4/35 ) 5. The result of BK virus (SV40 antigen) immunostaining is negative. | N/A | N/A | FK-506 dose reduction |
| 63 | SPK | Kidney | No | N/A | Clinical | N/A | Pulse 2.5g |
| 64 | SPK | Kidney | No | N/A | Clinical | N/A | Thymoglobulin 8.3mg/kg |
| 65 | SPK | Kidney | No | N/A | Clinical | N/A | Pulse 2.25g |
| 66 | SPK | Pancreas | Yes | 1. Rare acinar inflammatory foci ( < 1 focus/lobule ). 2. No septal inflammation. 3. Equivocal venular endothelialitis or ductitis. 4. Suspicious for acute T-cell mediated rejection. | borderline aTCMR | N/A | Pulse 1.5g |
| 67 | SPK | Kidney | No | N/A | Clinical | N/A | Pulse 2g |
| 68 | SPK | Kidney | No | N/A | Clinical | N/A | Pulse 750mg |
| 69 | SPK | Kidney | No | N/A | Clinical | N/A | Pulse 2.75g |
| 70 | SPK | Kidney | No | N/A | Clinical | N/A | TPE 3 times/Pulse 1.5g/Rituximab 200mg |
| 71 | SPLK | Pancreas | Yes | 1. Active septal inflammation with venulitis and ductitis 2. Multifocal acinar inflammation ( ~5 foci/lobule ) with spotty acinar cell injury 3. Mild intimal arteritis, consistent with acute T-cell mediated rejection, grade II. | aTCMR | II | Pulse 2.5g/Thymoglobulin 1mg/kg |
| 72 | SPK | Kidney | Yes | 1. Acute T cell-mediated rejection, grade (IA). 2. Acute thrombotic microangiopathy with mild glomerulitis. 3. Trivial interstitial fibrosis and tubular atrophy.  4. The result of BK virus (SV40 antigen) immunostaining is negative, supporting the diagnosis. | aTCMR | IA | TPE 4 times/Pulse 2g/FK-506 changed to cyclosporine |
| 73 | SPK | Kidney | Yes | 1. C4d immunonegativity with acute and chronic microcirculation injury, suspicious for chronic active antibody-mediated rejection. 2. Chronic active T cell-mediated rejection, grade IA . 3. Moderate interstitial fibrosis and tubular atrophy. 4. Focal global ( 8/26 ) and focal segmental ( 4/26 ) glomerular sclerosis, consistent with focal segmental glomerulosclerosis, probably secondary. 5. The result of BK virus (SV40 antigen) immunostaining is negative. | caTCMR/caABMR | IA | TPE 3 times/Pulse 2g/Rituximab 100mg/IVIG 600mg/kg, |
| 74 | SPK | Kidney | Yes | 1. Acute T cell-mediated rejection, grade IB. 2. No interstitial fibrosis and tubular atrophy. 3. Minimal glomerular lesion, consistent with IgA nephropathy Haas classification, subclass I, with 1) focal global ( 2/31 ) glomerular sclerosis, 2) no crescent formation, 3) oxford classification: M0, E0, S0, T0, C0. 4. The result of BK virus (SV40 antigen) immunostaining is negative, supporting the diagnosis. | aTCMR | IB | Pulse 3g |
| 75 | SPK | Kidney | Yes | 1. Mild tubulitis, suspicious of acute T-cell-mediated rejection. 2. Chronic active T-cell-mediated rejection. 3. Moderate interstitial fibrosis and tubular atrophy. 4. No glomerular sclerosis ( 0/11 ). 5. Results of immunohistochemical stainings: BK virus, (-); CD20, (+); CD56, (+). | borderline aTCMR/caTCMR | N/A | Pulse 1.5g |
| 76 | SPK | Kidney | No | N/A | Clinical | N/A | Pulse 750mg |
| 77 | SPK | Kidney | No | N/A | Clinical | N/A | Thymoglobulin 5mg/k |
| 78 | SPK | Pancreas | Yes | 1. Minimal septal inflammation 2. Minimal acinar inflammation 3. Mild allograft fibrosis, suggestive of acute T-cell-mediated rejection, consistent with chronic allograft rejection, stage I. | aTCMR | I | Thymoglobuin 4.5mg/kg |
| 79 | SPK | Kidney | No | N/A | Clinical | N/A | Pulse 3g |
| 80 | SPK | Kidney | Yes | 1. Moderate interstitial fibrosis and tubular atrophy. 2. Polyomavirus nephropathy, class 2. 3. Focal global and glomerular sclerosis ( 2/10 ). 4. The result of BK virus (SV40 antigen) immunostaining is positive, supporting the diagnosis. | N/A | N/A | FK-506 changed to cyclosporine |
| 81 | SPLK | Pancreas | Yes | 1. Septal inflammation with septal venulitis. 2. Mild acinar inflammation. 3. No C4d staining in interacinar capillaries. 4. Mild graft fibrosis, consistent with acute T-cell mediated rejection, grade I, consistent with chronic allograft rejection, stage I. | aTCMR | I | Thymoglobulin 4.7mg/kg |
| 82 | SPK | Kidney | No | N/A | Clinical | N/A | Pulse 2g |
| 83 | SPK | Kidney | Yes | 1. Acute T cell-mediated rejection, grade IA. 2. Mild interstitial fibrosis and tubular atrophy. 3. Focal global glomerular sclerosis ( 1/42 ). ( See note ) 4. The result of BK virus (SV40 antigen) immunostaining is negative, supporting the diagnosis. | aTCMR | IA | Pulse 2.5g |
| 84 | SPK | Kidney | Yes | 1. Acute tubular necrosis with tubular epithelial vacuolization and arteriolar hyaline change.  2. Suspicious (Borderline) for acute T cell-mediated rejection. 3. Ischemic wrinkling of glomeruli. 4. Focal scar in medulla. 5. Mild interstitial fibrosis and tubular atrophy. 6. Focal global ( 2/37 ) and focal segmental ( 2/37 ) glomerular sclerosis, consistent with focal glomerular sclerosis, probably secondary. 7. The result of BK virus (SV40 antigen) immunostaining is negative. | borderline aTCMR | N/A | Pulse 1.5g |
| 85 | SPK | Pancreas | Yes | 1. Minimal septal inflammation 2. Septal venulitis 3. Minimal acinar inflammation 4. No C4d-staining in interacinar capillaries 5. Minimal graft fibrosis, consistent with acute T-cell mediated rejection, grade I. | aTCMR | I | Observation |
| 86 | SPLK | Kidney | No | N/A | Clinical | N/A | Pulse 2.5g |
| 87 | SPK | Kidney | No | N/A | Clinical | N/A | Pulse 2g |
| 88 | SPK | Kidney | Yes | 1. Chronic active T cell-mediated rejection, grade IB (The chronic active T cell-mediated rejection, grade IB includes features of acute T cell-mediated rejection, grade IB.) 2. Mild interstitial fibrosis and tubular atrophy. 3. Focal global glomerular sclerosis ( 1/30 ).  4. The result of BK virus (SV40 antigen) immunostaining is negative, supporting the diagnosis. | aTCMR/caTCMR | IB | Pulse 2.5g |
| 89 | SPK | Kidney | Yes | 1. C4d immunopositivity with acute and chronic microcirculation injury, suspicious for chronic active antibody-mediated rejection. (The chronic active T cell-mediated rejection, grade IB includes features of suspicious (borderline) for acute T cell-mediated rejection and acute T cell-mediated rejection, grade IB) 2. Chronic active T cell-mediated rejection, grade IB. 3. Moderate interstitial fibrosis and tubular atrophy. 4. Focal global glomerular sclerosis ( 1/60 ). 5. The result of BK virus (SV40 antigen) immunostaining is negative. | caABMR/caTCMR | IB | Thymoglobulin 4.3mg/kg & IVIG 600mg/kg |
| 90 | SPK | Pancreas | Yes | 1. Moderate septal inflammation. 2. Venulitis and ductitis. 3. Multifocal acinar inflammation. 4. Mild graft fibrosis. 5. No C4d staining in interacinar capillaries, consistent with acute T-cell mediated rejection, grade II, consistent with chronic allograft rejection stage I. | aTCMR | II | Thymoglobulin 3.96mg/kg |
| 91 | SPK | Kidney | Yes | 1. Acute tubular injury and ischemic wrinkling of glomerular capillary wall, suggestive of ischemic injury. 2. Mild interstitial fibrosis and tubular atrophy. 3. No glomerular sclerosis ( 0/25 ). | N/A | N/A | FK-506 dose reduction |
| 92 | SPLK | Kidney | Yes | 1. Suspicious (Borderline) for acute T cell-mediated rejection. 2. Mild interstitial fibrosis and tubular atrophy. 3. No glomerular sclerosis ( 0/20 )   4. The result of BK virus (SV40 antigen) immunostaining is negative. | borderline aTCMR | N/A | Pulse 1.5g |
| 93 | SPK | Kidney | Yes | 1. Suspicious (Borderline) for acute T cell-mediated rejection. 2. C4d staining without evidence of antibody-mediated rejection. 3. Mild interstitial fibrosis and tubular atrophy. 4. Focal global glomerular sclerosis ( 1/48 ). ( See note ) 5. The result of BK virus (SV40 antigen) immunostaining is negative. | borderline aTCMR | N/A | Pulse 1.5g |
| 94 | SPK | Kidney | Yes | 1. No evidence of active rejection. 2. Mild interstitial fibrosis and tubular atrophy. 3. No glomerular sclerosis ( 0/26 ). ( See note ) 4. Suggestive of tubular vacuolization, rule out acute drug/toxic tubular injury. 5. The result of BK virus (SV40 antigen) immunostaining is negative. | N/A | N/A | Observation |
| 95 | SPLK | Kidney | Yes | . Acute tubulointerstitial nephritis. 2. Acute thrombotic microangiopathy with focal microthrombi in glomerular capillaries and arterioles. 3. Mild interstitial fibrosis and tubular atrophy. 4. Focal global glomerular sclerosis ( 6/31 ) 5. The result of BK virus (SV40 antigen) immunostaining is negative. | N/A | N/A | TPE 2 times/FK-506 dose reduction |
| 96 | SPK | Kidney | Yes | 1. Acute tubulointerstitial nephritis. 2. No evidence of rejection. 3. No interstitial fibrosis and tubular atrophy. 4. No glomerular sclerosis ( 0/20 ).  5. The result of BK virus (SV40 antigen) immunostaining is negative. | N/A | N/A | Pulse 2.5g |
| 97 | SPLK | Pancreas | Yes | 1. Mild septal inflammation. 2. Mild venulitis. 3. Mild acinar inflammation. 4. Mild graft fibrosis ( < 30 % ), consistent with acute T-cell mediated rejection, grade I, chronic allograft rejection, grade I. | aTCMR | I | Pulse 1g |
| 98 | SPK | Kidney | Yes | 1. C4d staining with uncertain significance (C4d-immunopositivity, along with donor specific antibody, may represent impending antibody-mediated rejection.) 2. Diffuse isometric vacuolization of tubular epithelial cells. 3. No interstitial fibrosis and tubular atrophy. 4. No glomerular sclerosis ( 0/27 ). 5. The result of BK virus (SV40 antigen) immunostaining is negative. | aABMR | N/A | TPE 4 times/Rituximab 100mg |

SPK, simultaneous pancreas-kidney transplant; SPLK, simultaneous deceased donor pancreas and living donor kidney transplant; BK virus, polyomavirus; aTCMR, acute T-cell mediated rejection; caTCMR, chronic acute T-cell mediated rejection; aABMR, acute/active antibody-mediated rejection; caABMR, chronic active antibody-mediated rejection; TPE, therapeutic plasma exchange; IVIG, intravenous immunoglobulin
